# Unexpected Impact of a Hepatitis C Virus Inhibitor on 17β-Estradiol Signaling in Breast Cancer

**DOI:** 10.3390/ijms21103418

**Published:** 2020-05-12

**Authors:** Stefania Bartoloni, Stefano Leone, Filippo Acconcia

**Affiliations:** Department of Sciences, Section Biomedical Sciences and Technology, University Roma Tre, Viale Guglielmo Marconi, 446, I-00146 Rome, Italy; stefania.bartoloni2@uniroma3.it (S.B.); stefano.leone@uniroma3.it (S.L.)

**Keywords:** 17β-estradiol, estrogen receptor α, telaprevir, breast cancer

## Abstract

17β-Estradiol (E2) controls diverse physiological processes, including cell proliferation, through its binding to estrogen receptor α (ERα). E2:ERα signaling depends on both the receptor subcellular localization (e.g., nucleus, plasma membrane) and intracellular ERα abundance. Indeed, the control of ERα levels is necessary for the effects of E2, and E2 itself induces ERα degradation and cell proliferation in parallel. Thus, the modulation of intracellular ERα levels is a critical parameter for E2-induced cell proliferation. Therefore, we used this parameter as a bait to identify compounds that influence ERα levels and E2-dependent proliferation in breast cancer (BC) cells from a library of Food and Drug Administration (FDA)-approved drugs. We found that telaprevir (Tel) reduces ERα levels and inhibits BC cell proliferation. Tel is an inhibitor of the hepatitis C virus (HCV) NS3/4A serine protease, but its effect on E2:ERα signaling has not been investigated. Here, for the first time, we analyzed the effects of Tel on intracellular ERα levels and E2:ERα signaling to cell proliferation in different ERα-expressing BC cell lines. Overall, our findings demonstrate that Tel reduces intracellular ERα levels, deregulates E2:ERα signaling and inhibits E2-induced proliferation in BC cells and suggest the potential drug repurposing of Tel for the treatment of BC.

## 1. Introduction

The hormone 17β-estradiol (E2), the most effective estrogen, regulates a plethora of physiological effects by binding estrogen receptor α (ERα) [1,2]. ERα, which belongs to the nuclear receptor superfamily, acts as a ligand-activated transcription factor to drive E2-responsive gene expression through multiple mechanisms (i.e., nuclear and extranuclear signaling) [2,3,4,5,6,7,8,9].

Moreover, E2 action is a function of not only the subcellular localization but also the intracellular levels of ERα [3,10]. The ERα protein exhibits a high turnover rate, and intracellular receptor levels change over time. Change in the ERα content is necessary to drive E2-dependent cell functions and regulate cell responsiveness to this hormone. Indeed, E2 itself controls the ERα content, simultaneously triggering receptor degradation and inducing cell proliferation [3,10]. Thus, the modulation of intracellular ERα levels is a critical parameter for E2 signaling [3,9,11]. Hence, factors that deregulate the physiological control of intracellular ERα levels can potentially inhibit E2 signaling and downstream effects, such as cell proliferation. Accordingly, synthetic ERα ligands (e.g., fulvestrant [ICI182,780-ICI] and tamoxifen) alter intracellular ERα levels and inhibit E2-dependent breast cancer (BC) cell proliferation [10,12]. Molecules that do not bind ERα (e.g., carfilzomib and emetine) also influence the ERα abundance and prevent E2-induced BC cell proliferation [13,14]. In addition, the inhibition or depletion of proteins belonging to pathways unrelated to E2:ERα signaling (e.g., metabolic and endocytic proteins) blocks BC cell proliferation, changing the cellular ERα content [11,15,16,17,18,19]. Thus, the modulation of intracellular ERα levels and E2-dependent BC cell proliferation are interrelated.

Based on this evidence, our laboratory recently demonstrated that modulation of the ERα content can be used as a parameter to identify compounds that can alter ERα levels and prevent E2-dependent BC cell proliferation [3,13,14,15,20]. Thus, a library of Food and Drug Administration (FDA)-approved drugs was applied to ERα-expressing ductal carcinoma cells (MCF-7), and seven compounds were found to influence the ERα content and cell proliferation [14]. The effects of one such compound, telaprevir (Tel), on these processes have never been investigated. Tel is a reversible inhibitor of the hepatitis C virus (HCV) NS3/4A serine protease approved by the FDA for the treatment of patients with genotype 1 HCV infection, in combination with pegylated interferon and ribavirin [21,22].

Here, we aimed to validate and characterize the impact of Tel on E2:ERα signaling to BC cell proliferation.

## 2. Results

### 2.1. Telaprevir Affects Intracellular ERα Levels

**Figure 1:**
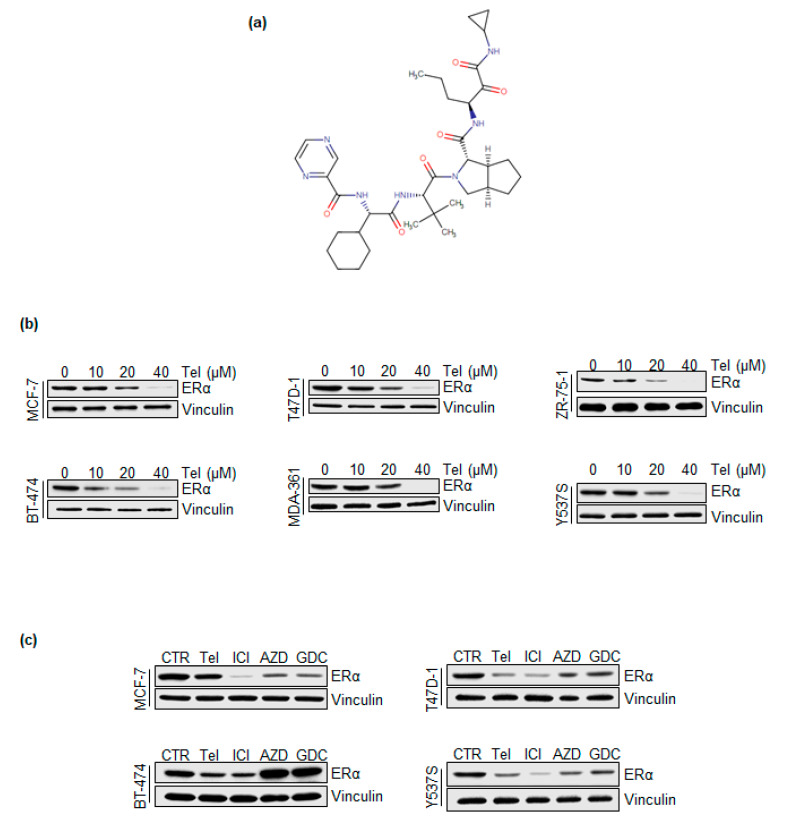
The effect of telaprevir on ERα intracellular levels. (**a**) Schematic of the telaprevir chemical formula as depicted in DrugBank (n.: DB05521). (**b**) Western blotting analysis of ERα cellular levels in MCF-7, T47D-1, ZR-75-1, BT-474, MDA-MB-361 and Y537S cells treated with different doses of telaprevir (Tel) for 24 h. The loading control was done by evaluating vinculin expression in the same filter. (**c**) Western blotting analysis of ERα expression levels in MCF-7, T47D-1, BT-474 and Y537S cells treated with Tel (20 µM), fulvestrant (ICI 100 nM), AZD9496 (AZD 100 nM) and GDC-0810 (GDC 100 nM) for 24 h. The loading control was done by evaluating vinculin expression in the same filter. Panels a and b show representative blots from at least three independent experiments.

To identify new compounds that can influence intracellular ERα levels and E2-dependent proliferation in BC cells, a screen of FDA-approved drugs was performed in our laboratory [14]. The results of the screen suggested that telaprevir (Tel) (Figure 1a) reduces intracellular ERα levels and prevents basal and E2-induced proliferation in MCF-7 cells. Therefore, initial experiments were undertaken to validate the effect of Tel on the ERα content in different ERα-expressing BC cell lines [MCF-7, T47D-1, ZR-75–1, BT-474, MDA-MB-361 and Y537S-ERα mutant-expressing MCF-7 (Y537S) cells]. For this purpose, cells were treated with increasing doses of Tel (10 to 40 µM) for 24 h. As shown in Figure 1b, Tel reduced intracellular ERα levels in a dose-dependent manner in each cell line tested. In particular, 20 µM Tel significantly reduced intracellular ERα levels in most of the cell lines, although the lowest concentration of Tel at which a reduction in ERα was observed differed between the tested cell lines.

In addition, the Tel-mediated reduction in ERα levels was compared to the effects of three different selective estrogen receptor downregulators (SERDs): fulvestrant (ICI 182,780-ICI), AZD9496 (AZD) and brilanestrant (GDC-0810-GDC) [23,24]. For this purpose, MCF-7, T47D-1, BT-474 and Y537S cells were treated with Tel (20 µM), ICI (100 nM), AZD (100 nM) and GDC (100 nM) for 24 h. The results (Figure 1c) indicate that Tel and ICI reduced ERα levels in each cell line tested, as expected. Interestingly, the ability of Tel to reduce the receptor content in T47D-1 and BT-474 cells was similar to that of ICI. Moreover, AZD and GDC induced a decrease in ERα content in MCF-7, T47D-1 and Y537S cells, although the effects of these antiestrogens were not observed in BT-474 cells. Notably, Tel reduced the ERα content in T47D-1 and BT-474 cells to a greater extent than AZD or GDC.

Overall, these findings demonstrate that Tel influences the control of intracellular ERα levels and that the ability of Tel to reduce the ERα content of BC cells is similar to that of SERDs.

### 2.2. Telaprevir-Dependent Mechanism for the Control of ERα Levels

**Figure 2:**
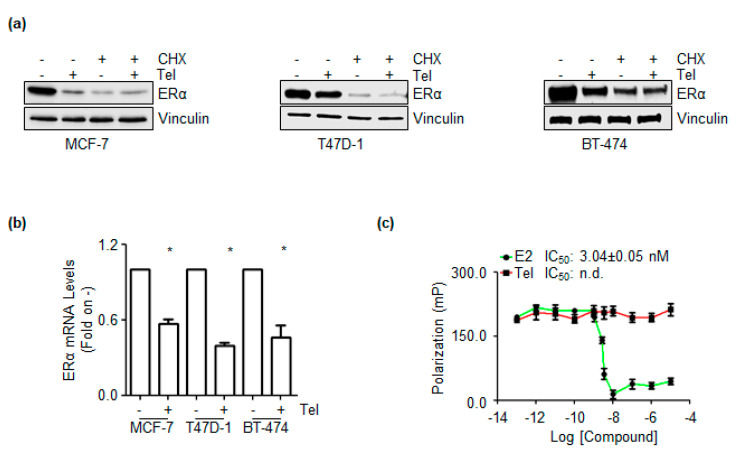
The mechanism of telaprevir-dependent ERα reduction. (**a**) Western blotting analysis of pre-formed ERα levels in MCF-7, T47D-1 and BT-474 cells pre-treated with cycloheximide (CHX 1 µg/mL) for 6 h and then treated with telaprevir (Tel 20 µM) for 24 h. The loading control was done by evaluating vinculin expression in the same filter. Panels show representative blots from at least three independent experiments. (**b**) In vitro ERα competitive binding assays for telaprevir (Tel–red) and 17β-estradiol (E2–green) performed at different doses of the compounds and using a florescent E2 as tracer. Inhibitor concentration 50 (IC_50_–nM) is indicated in the panel for each compound. The experiment was performed twice in quintuplicate. (**c**) RT-qPCR analysis of ERα mRNA levels in MCF-7, T47D-1 and BT-474 cells treated with Tel (20 µM) for 24 h. ERα mRNA expression was normalized to the GAPDH mRNA expression. * indicates significant differences with respect to the control sample (−). Data are the mean ± standard deviations with a *p* value < 0.01. All experiments were performed in triplicate.

The intracellular levels of ERα in pools of both preformed and neosynthesized ERα are controlled [25]. Since Tel influences ERα abundance (Figure 1), the effect of this compound on the pool of ERα already produced in the cell (i.e., preformed ERα) was investigated in MCF-7, T47D-1,BT-474 cells as well as in Y537S cells (Supplementary Figure S1b). Thus, cells were pretreated with the protein synthesis inhibitor cycloheximide (CHX, 1 µg/mL) for 6 h before 24 h of Tel (20 µM) treatment. The results showed that Tel and CHX reduced ERα levels, as expected. Interestingly, Tel did not influence the CHX-dependent reduction in intracellular ERα levels in each cell line tested (Figure 2a). These data suggest that Tel does not affect preformed ERα. Next, experiments were performed to assess the ability of Tel to alter ERα mRNA levels and hence the levels of newly produced ERα by mRNA translation (i.e., neosynthesized ERα) [10,25]. As shown in Figure 2b, Tel reduced ERα mRNA levels in MCF-7, T47D-1 and BT-474 cells. Taken together, these findings suggest that Tel controls the ERα content at the transcriptional level, triggering a decrease in ERα mRNA expression in MCF-7, T47D-1 and BT-474 cells.

However, these results did not exclude the possibility that Tel can bind ERα. Therefore, the capability of Tel to bind ERα was analyzed through an in vitro fluorescence polarization-based competitive binding assay performed at room temperature and under steady-state conditions (i.e., measurement of the binding was performed at 2 h). Figure 2c illustrates that E2 displaced the fluorescent ligand mimicking E2 from ERα and bound the receptor with an IC_50_ (i.e., K_d_) of approximately 3 nM. Notably, the measured K_d_ of E2 towards ERα was in the range of that measured under different conditions and with different techniques [3,26]. Conversely, Tel did not induce displacement of the fluorescent ligand, indicating that Tel could not bind ERα in vitro.

### 2.3. Effect of Telaprevir on ERα Transcriptional Activity

**Figure 3:**
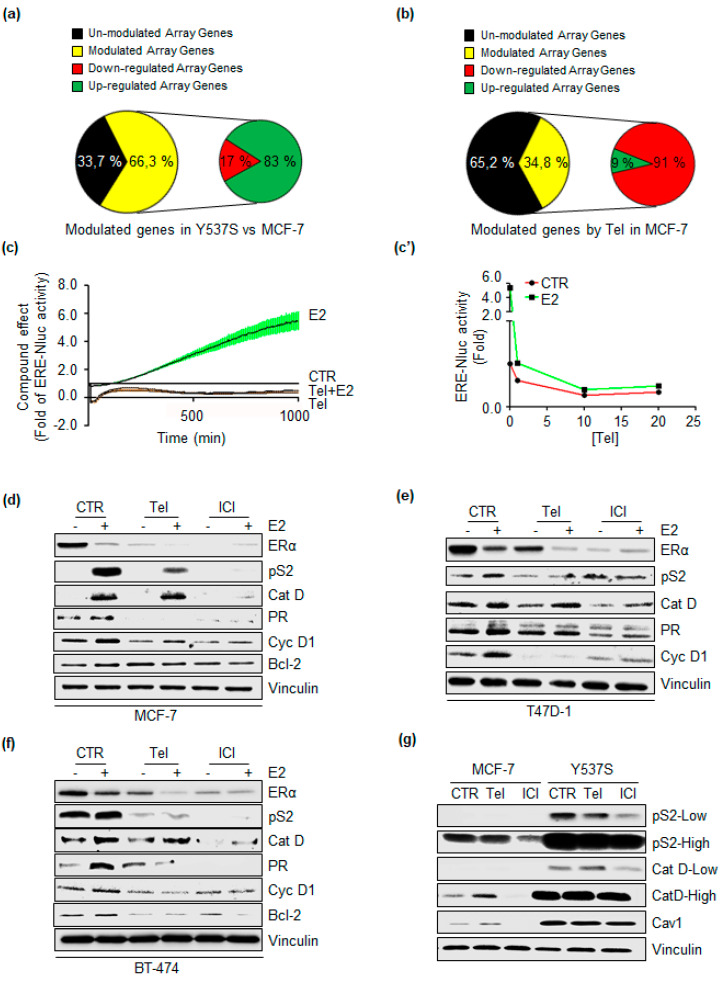
The effect of Telaprevir on E2:ERα nuclear signaling. (**a**) Pie diagrams representing the percentage of the array genes modulated in Y537S compared to MCF-7 cells and (**b**) pie diagrams depicting the percentage of the array genes modulated by 24 h of telaprevir (Tel 20 µM) treatment in MCF-7 cells. (**c**) Time-dependent profile of ERE-NLuc activity detected in MCF-7 ERE-NLuc cells treated with Tel (20 µM) and 17β-estradiol (E2 10 nM). (**c’**) Profile of ERE-NLuc activity detected in MCF-7 ERE-NLuc cells treated with different doses of Tel in the absence and in the presence of E2 (10 nM) and detected after 24 h of compound administration. (**d–f**) Western blotting analysis of ERα, presenilin 2 (pS2), cathepsin D (Cat D), progesterone receptor (PR), cyclin D1 (Cyc D1) and Bcl-2 expression in MCF-7, T47D-1 and BT-474 cells pre-treated with Tel (20 µM) and fulvestrant (ICI 100 nM) for 24 h before 24 h of E2 (10 nM) treatment. The loading control was done by evaluating vinculin expression on the same filter. (**g**) Western blotting analysis of pS2, Cat D and caveolin 1 (Cav 1) protein levels in Y537S cells compared to MCF-7 cells. Cells were treated with Tel (20 µM) and ICI (100 nM) for 24 h. The loading control was done by evaluating vinculin expression on the same filter. Panels d, e, f and g show representative blots from at least three independent experiments.

ERα degradation is intrinsically connected with the transcriptional activity of the receptor [27,28]. Thus, the impact of Tel on ERα transcriptional activity was analyzed. Initial experiments were performed to evaluate the influence of Tel on ERα target gene expression through RT-qPCR-based E2-sensitive gene array analysis. Initially, the quality of the assay was tested by comparing MCF-7 cells and mutant ERα-expressing MCF-7 (Y537S) cells. As Y537S-ERα is constitutively activated in the absence of E2 [29], Y537S cells were used as a model to measure E2-induced gene expression. The pie diagrams in Figure 3a show that 66.3% (yellow) of the array genes were significantly modulated in Y537S cells compared to MCF-7 cells and that 83% (green) of these genes were upregulated in Y537S cells. Among them were trefoil factor 1 (TFF1-pS2), cathepsin D (Cat D) and caveolin 1 (Cav 1), as expected [29]. Thus, the assay effectively gauged E2:ERα signaling. Next, the effect of Tel was analyzed in MCF-7 cells treated for 24 h with the antiviral. As shown in Figure 3b, Tel modulated 34.8% (yellow) of the genes in the array. Interestingly, 91% (red) of the modulated genes were downregulated by Tel, suggesting that the compound prevents ERα transcriptional activity.

To support this observation, the effect of Tel was next analyzed by measuring the real-time kinetics of ERα transcriptional activity in MCF-7 cells stably transfected with an E2-responsive ERE-nanoluciferase (NLuc)-PEST reporter gene construct (MCF-7 ERE-NLuc) [30]. The results reported in Figure 3c show that E2 (10 nM) induced a time-dependent increase in ERE-NLuc activity, as expected [30]. Notably, Tel blocked basal and E2-induced ERE-NLuc activity in a time-dependent manner (Figure 3c). Dose response analyses were also performed, which showed an effect after 24 h of treatment (Figure 3c’). Therefore, this evidence indicates that Tel inhibits the transcriptional activity of the receptor.

Consequently, the ability of Tel to alter the expression of estrogen-responsive element (ERE)-containing genes (e.g., presenilin2 [pS2], cathepsin D [Cat D] and progesterone receptor [PR]) and non-ERE-containing genes (e.g., Bcl-2 and cyclin D1 [Cyc D1]) was evaluated in MCF-7, T47D-1 and BT-474 cells. Cells were treated with Tel (20 µM) and ICI (100 nM, as an internal control) in the absence and presence of E2 (10 nM). Notably, E2-dependent ERα degradation in each cell line was enhanced in the presence of Tel, while Tel administration reduced the receptor content (Figure 3d–f). These results support the notion that Tel does not bind ERα. In addition, Tel prevented E2-induced accumulation of pS2, PR and Cyc D1 in MCF-7, T47D-1 and BT-474 cells (Figure 3d–f) and blocked E2-dependent Bcl-2 overexpression in MCF-7 and BT-474 cells (Figure 3d,f). However, the ability of E2 to trigger Cat D expression was not inhibited by Tel (Figure 3d–f). Moreover, the results indicated that Tel increased the basal protein level of Bcl-2 in MCF-7 cells (Figure 3d). These last observations suggest that a mechanism unrelated to ERα is involved in the regulation of Cat D and Bcl-2 expression.

Finally, the protein levels of three genes (e.g., pS2, Cat, Cav 1), whose expression was upregulated in Y537S cells [29], were analyzed to confirm the impact of Tel in preventing ERα transcriptional activity. Consistent with the array gene analysis (Figure 3a), pS2, Cat D and Cav 1 expression was upregulated in Y537S cells compared to MCF-7 cells (Figure 3g). Furthermore, Tel reduced pS2 levels in Y537S cells but did not influence Cat D or Cav 1 expression in this cell line (Figure 3g), thus supporting the possibility of an alternative mechanism for the control of their expression.

Overall, these results demonstrate that Tel blocks ERα transcriptional activity and suggest that a Tel-dependent reduction in intracellular ERα levels affects, at least in part, the expression of E2:ERα target genes.

### 2.4. Effect of Telaprevir on E2-Dependent Breast Cancer Cell Proliferation

E2-dependent activation of ERα transcriptional activity promotes DNA synthesis, cell cycle progression and cell proliferation in BC cells [8,31]. Therefore, the impact of Tel on E2-dependent DNA synthesis and cell cycle progression was studied through a bromodeoxyuridine (BrdU) incorporation assay and cell cycle analysis, respectively, in MCF-7, T47D-1 and BT-474 cells.

**Figure 4:**
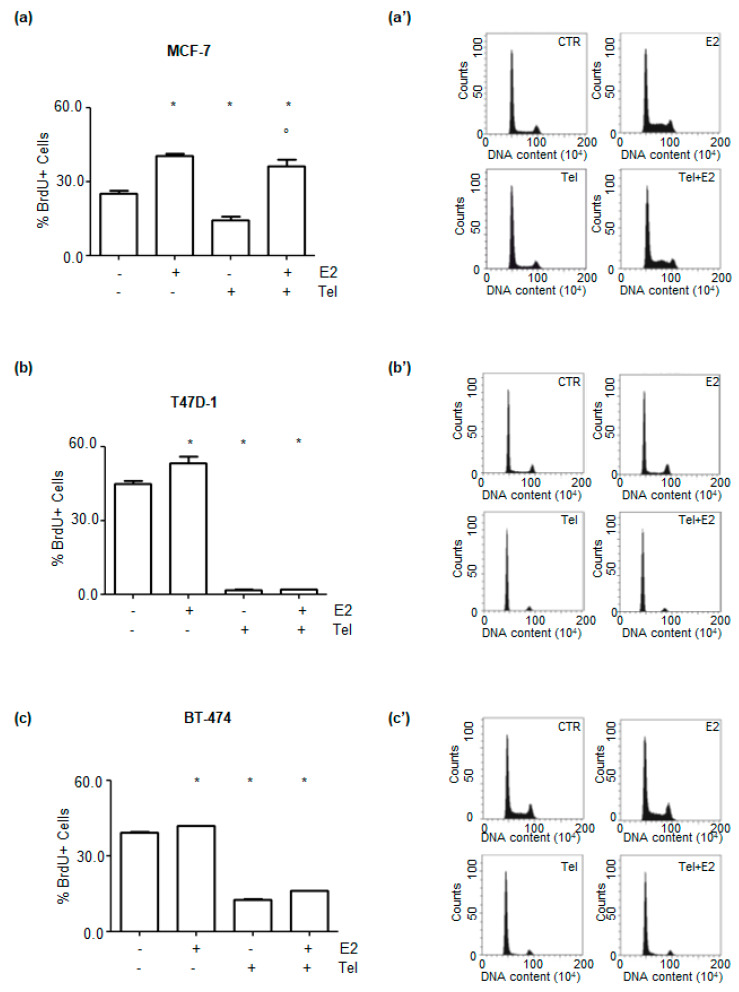
The effect of telaprevir on E2-dependent DNA synthesis and cell cycle progression. (**a–c**) bromodeoxyuridine (BrdU) incorporation assay and (**a’–c’**) cell cycle analysis in MCF-7, T47D-1 and BT-474 cells. Cells were pre-treated with telaprevir (Tel 10 µM in MCF-7 cells, 20 µM in T47D-1 and BT-474 cells) for 24 h before 24 h of 17β-estradiol (E2 10 nM) treatment. * indicates significant differences with respect to control sample (−,−), ° indicated significant differences with respect to the corresponding E2 CTR sample (+,−). Data are the mean ± standard deviations with a *p* value < 0.01.

As expected, E2 triggered BrdU incorporation in each cell line tested (Figure 4a–c). Tel prevented basal and E2-induced BrdU incorporation in T47D-1 and BT-474 cells (Figure 4b,c), while in MCF-7 cells, Tel reduced basal BrdU incorporation but could not prevent the E2-induced increase in DNA synthesis (Figure 4a). Furthermore, cell cycle analysis (Figure 4a’–c’) indicated that E2 treatment increased the number of MCF-7, T47D-1 and BT-474 cells in the S and G2 phases of the cell cycle. In contrast, Tel led to the accumulation of cells in the G1 phase of the cell cycle in each cell line. Moreover, Tel prevented E2-dependent accumulation of T47D-1 and BT-474 cells in the S and G2 phases of the cell cycle but did not affect the accumulation of MCF-7 cells in the S and G2 phases. Therefore, these findings suggest that Tel could interfere with the proliferative ability of the tested cell lines.

**Figure 5:**
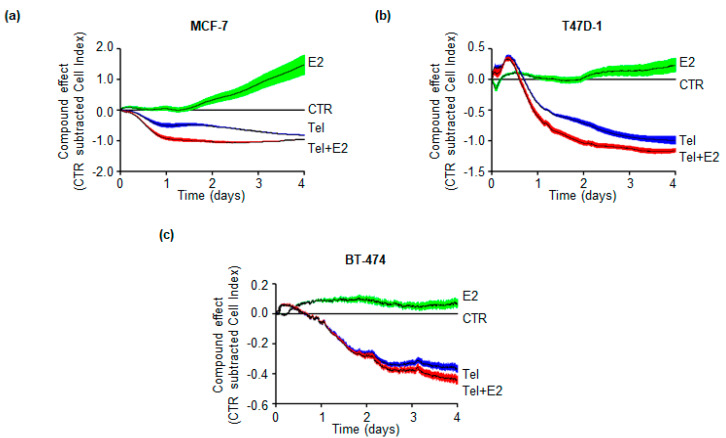
The effect of telaprevir on breast cancer cell proliferation. Real-time growth curves in (**a**) MCF-7, (**b**) T47D-1 and (**c**) BT-474 treated with telaprevir (Tel 20 µM) in the absence (blue line) and in the presence (red line) of 17β-estradiol (E2 10 nM) and with E2 alone (10 nM green line) for the indicated time points. The graphs show Tel and E2 effect on the cell index (i.e., cell number), which is detected with the xCelligence DP device and calculated at each time point with respect to control sample (CTR grey line). Each sample was measured in quadruplicate. For details, please see material and methods section.

In turn, real-time growth curve analysis was conducted in MCF-7, T47D-1 and BT-474 cells as previously reported [30]. As shown by the results (Figure 5a–c), E2 promoted the proliferation of MCF-7, T47D-1 and BT-474 cells, while Tel treatment significantly reduced basal and E2-induced proliferation in each cell line. Notably, Tel in the presence of E2 reduced cell proliferation to a higher extent than Tel treatment alone (Figure 5a–c).

Taken together, these data indicate that Tel blocks basal and E2-induced BC cell proliferation.

## 3. Discussion

The primary aim of this work was to characterize the effect of the antiviral Tel on the regulation of intracellular ERα levels and on E2:ERα signaling in BC cell proliferation. E2 is a mitogen for BC, and ERα is the main driver of E2-dependent proliferation in approximately 70% of all cases of BC [8,9,12,31]. Moreover, the cellular effects of E2 are correlated with hormone-dependent regulation of the intracellular ERα content. Indeed, simultaneously E2 reduces ERα levels and induces cell proliferation. Therefore, the control of intracellular ERα abundance is required for proper cell responsiveness to E2 [3,8,9,10,12,31,32].

Interestingly, accumulating data suggest that factors that deregulate the physiological control of intracellular ERα levels can inhibit cell proliferation elicited by E2. In turn, synthetic ERα ligands (e.g., fulvestrant and tamoxifen) and molecules that do not bind the receptor (e.g., chloroquine, emetine and carfilzomib) alter intracellular ERα levels and inhibit BC cell proliferation [3,12]. Furthermore, the inhibition or depletion of proteins belonging to pathways unrelated to E2:ERα signaling (e.g., metabolic and endocytic proteins) also prevent BC cell proliferation by changing the ERα content [11,15,16,17,18,19,33]. Therefore, the modulation of intracellular ERα levels is a sensitive parameter for E2-dependent cell proliferation. In this respect, it was recently proposed that the modulation of intracellular ERα levels could be used as a bait to identify compounds that can influence the ERα content, and in turn, cell proliferation [3]. These compounds that directly or indirectly affect the balance between the modulation of ERα levels and E2-dependent proliferation were grouped in a supercategory and defined as selective modulators of ERα levels and degradation (e.m.eral.d.s) [3]. Based on this proof of concept study, we screened a library of FDA-approved drugs in ERα-expressing ductal carcinoma cells (MCF-7) [14] and found the unsuspected role of Tel as a compound that reduces ERα levels and prevents basal and E2-induced cell proliferation. Tel is an inhibitor of the hepatitis C virus (HCV) NS3/4A serine protease [21], but its effect on E2:ERα signaling in cell proliferation has never been investigated.

Here, we demonstrate for the first time that Tel reduces ERα levels in six different ERα-expressing BC cell lines, acting as models of primary (i.e., MCF-7, T47D-1, and ZR-75-1 cells) and metastatic (i.e., BT-474, MDA-MB-361 and Y537S-ERα mutant-expressing MCF-7 [Y537S] cells) BC [3]. Treatment with 20 µM Tel results in a significant decrease in the ERα content of MCF-7, T47D-1, ZR-75-1, BT-474 and Y537S cells but not MDA-MB-362 cells, but this effect was observed when the compound was applied at higher doses in this latter cell line. Although we observed the effect of Tel at a micromolar concentration, it is important to note that this relatively high dose is in the range of the plasma concentration of Tel reached after administration of Tel at therapeutic doses [34]. Moreover, the effect of Tel on ERα protein levels was observed under both growth (Figure 1b) and starvation (Supplementary Figure S1a) conditions. Interestingly, Tel reduces ERα levels at lower concentrations under starvation conditions than under growth conditions. This phenomenon is probably due to the different growth rates of the cells in different cell culture media. Notably, this observation suggests not only that Tel activity is related to the cell cycle but also that Tel is more effective when used in combination with drugs that induce cell cycle arrest in G1 phase. Accordingly, we observed that Tel blocks the cells in G1 phase of the cell cycle (see also below). In addition, the fact that the effect of Tel on ERα degradation is generally comparable to that of the antiestrogens (i.e., competitive ERα antagonists that induce ERα degradation via the 26S proteasome [23,24]) fulvestrant (ICI182,780-ICI), AZD9496 (AZD) and brilanestrant (GDC-0810 GDC) in MCF-7, T47D-1, BT-474 and Y537S cells (Figure 1c) indicates that this drug could work in a manner similar to that of SERDs in BC cells.

However, the Tel-mediated reduction in ERα occurs at the posttranscriptional level, as Tel induces a decrease in ERα mRNA levels without binding the receptor (Figure 2) or affecting 26S proteasome activity (data not shown). We did not analyze the mechanism underlying the effect of Tel on ERα mRNA levels in this study; however, expression of the ERα gene (*ESR1*) is regulated by genetic and epigenetic mechanisms that in turn could be influenced by the antiviral compound [3,9]. *ESR1* is under the control of at least seven promoters, and different transcription factors control its differential expression in target tissues [3,9]. Interestingly, several lines of evidence suggest that ERα expression strongly correlates with the expression of two transcription factors, FOXA1 and GATA-3, in BC [35,36]. Notably, both the FOXA1 and GATA-3 genes were included in the E2-sensitive gene array, and we found that Tel reduces the levels of these genes (Figure 3b). Moreover, histone methylation regulates ERα mRNA synthesis, and the inhibition or siRNA-mediated depletion of histone methyltransferases (e.g., DOT1L and WHSC1) downregulated ERα expression in BC cells [37,38]. Therefore, a possible explanation for the Tel-mediated reduction in ERα is that Tel impairs the activity of transcription factors and/or epigenetic regulators involved in the control of ERα expression. However, it is important to note that Tel affects ERα levels in all the tested cell lines, suggesting a certain degree of specificity for Tel activity in BC cells.

Additionally, the presented data indicate that the Tel-mediated reduction in intracellular ERα levels partially turns off ERα target gene expression by preventing transcriptional activity of the receptor. RT-qPCR-based gene array analysis of ERα signaling revealed that E2-target gene downregulation prevails after Tel treatment, as 91% of the genes included in the array that are modulated by Tel were downregulated in MCF-7 cells (Figure 3b). We initially evaluated the quality of the test used to measure ERα nuclear signaling. As the Y537S mutation confers estrogen-independent activity to the receptor, we evaluated E2-induced target gene expression in genome-edited Y537S-ERα-expressing cells (Y537S) [29] in comparison with parental MCF-7 cells. We concluded that the assay effectively measures E2:ERα signaling because expression of the array genes was significantly upregulated in Y537S cells compared to MCF-7 cells (Figure 3a). Among the modulated genes were trefoil factor 1 (TFF1 or pS2), cathepsin D (Cat d) and caveolin 1 (Cav 1), which are known to be overexpressed in Y537S cells [29]. We also confirmed the overexpression of these genes in Y537S cells compared to MCF-7 cells by Western blotting (Figure 3g). Hence, we assumed that results of the array gene analysis clearly reflect the impact of the tested antiviral on ERα nuclear signaling. Accordingly, real-time evaluation of ERα transcriptional activity in MCF-7 ERE-NLuc cells [30] showed that Tel blocks basal and E2-induced ERα transcriptional activity in a time- and dose-dependent manner (Figure 3c,c’). Furthermore, the effect of Tel on ERα transcriptional activity also influences the ability of E2 to induce expression of ERE- and non-ERE-containing genes. In turn, Tel prevents E2-dependent pS2, Cat D and PR accumulation in MCF-7, T47D-1 and BT-474 cells and Bcl-2 upregulation in MCF-7 and BT-474 cells (Figure d–f). Notably, Tel also partially affects the activity of the Y537S-ERα mutant. The Y537S mutation, one of the most recurrent point mutations in *ESR1*, is linked to acquired resistance to endocrine therapy in metastatic BC [29,39,40,41]. Interestingly, Tel reduces pS2 expression in Y537S cells, even though Cav 1 expression is unresponsive to Tel and antiestrogen (i.e., ICI) treatment in this cell line (Figure 3g).

Nevertheless, E2 still induces Cat D expression in the presence of Tel in each cell line. In addition, Tel itself enhances basal Bcl-2 protein levels in MCF-7 cells, presumably as a compensatory response of the cells for survival [42]. The expression of Cat D and Bcl-2 may rely on a mechanism unrelated to ERα involving another type of estrogen receptor. Recently, G-protein coupled estrogen receptor 1 (GPER1, also known as GPR30) was discovered as a new E2 receptor, and its role in mediating cell responses to E2 has been studied both in vitro and in vivo. GPR1 is expressed in several BC cell lines and contributes to E2 and antiestrogen nuclear and extranuclear signaling in BC [43,44]. Thus, we cannot exclude that GPR1 is involved in Cat D and Bcl-2 expression in the presence of Tel, as evidence suggests the involvement of this receptor in transcriptional activation of E2-target genes (e.g., Cat D, Bcl-2 and Cyc D1) [43].

Tel activity blocks the mitogenic effects evoked by E2 in BC cells. In turn, Tel perturbs basal and E2-dependent DNA synthesis, cell cycle progression and proliferation in three different BC cell lines (i.e., MCF-7, T47D-1 and BT-474 cells) (Figure 4 and Figure 5). E2-dependent promotion of the G1 to S phase transition plays a critical role in BC pathogenesis. The action of E2 in cell cycle progression involves ERα-mediated cyclin D1 overexpression, cyclin-dependent kinase (CDK) activation and retinoblastoma (Rb) protein phosphorylation [1,31]. Therefore, targeting the ability of E2 to induce cell cycle progression is an important issue for BC treatment. In turn, antiestrogens (e.g., tamoxifen and fulvestrant) and selective inhibitors of CDK4/6 (e.g., Palbociclib) counteract E2 activity, triggering cell cycle arrest in G1 phase of the cell cycle in BC cells [12,31]. We report that Tel induces the accumulation of BC cells in G1 phase of the cell cycle also in response to E2 stimulation. This observation is consistent with the effect of the abovementioned compounds already used in clinics for the treatment of BC. Nonetheless, we found that Tel does not prevent E2-induced DNA synthesis or cell cycle progression in MCF-7 cells (Figure 4a), in contrast to the observed effects in T47D-1 and BT-474 cells. In this respect, it is tempting to speculate that the residual effect of E2 can be ascribed to the different levels of ERα in the different cell lines [45] after Tel administration. However, real-time growth curve analysis revealed that Tel inhibits both basal and E2-dependent proliferation of MCF-7, T47D-1 and BT-474 cells. Surprisingly, the antiproliferative effect of Tel is enhanced in the presence of E2. Thus, E2 may have a toxic effect on cells exposed to Tel. This situation could be due to the influence of Tel on pathways that protect cells against excessive proliferative and transcriptional stimuli induced by E2 [46,47]. From this perspective, ovarian function suppression and consequent premature menopause [48] would no longer be necessary for premenopausal women with ERα-positive BC who would receive Tel treatment. However, the selectivity of Tel for BC instead of different types of cancer as well as Tel-mediated toxicity in normal breast cells (e.g., MCF10a cells) requires further evaluation.

Another important issue to discuss concerns the molecular target of Tel in BC cells. Tel is a direct antiviral agent designed to target the HCV NS3/4A serine protease. Inhibition of the NS3/4A serine protease by Tel blocks cleavage of the genome-encoded polyprotein into mature viral proteins, preventing the generation of the machinery required for HCV replication [21]. Moreover, Tel activity also prevents NS3/4A-dependent proteolysis of cellular proteins (e.g., TRIF, MAVS and CARIF) involved in the host innate immune response, which leads to the inhibition of type-I interferon (INF) induction and consequent HCV escape from the immune system [49,50,51,52]. Given the essential role of type-I INFs as regulators of cancer immunosurveillance in BC, it might be interesting to evaluate whether Tel influences components of the IFN system in BC cells [53,54,55]. However, it has been reported that Tel is a substrate and inhibitor of P-glycoprotein (P-gp, ABCB1), which is an ATP-binding cassette transporter involved in ADME of a variety of drugs and implicated in multidrug resistance [56,57,58]. Therefore, the inhibitory potential of Tel against P-gp could enable clinically relevant drug interactions, which must be considered. In addition, to define a possible candidate molecular target for Tel in BC cells, we hypothesized that this antiviral affects eukaryotic protease activities since Tel is an inhibitor of a viral proteases. Therefore, we analyzed the impact of Tel on the three protease activities (i.e., chymotrypsin-like, caspase-like and trypsin-like) in the proteasome, the main protease-based system in the cell, in vitro. However, Tel has no effect (data not shown) on this cellular proteolytic system. Thus, the challenge of identifying cellular target(s) of Tel in BC cells remains an open question that needs to be addressed through different approaches (e.g., transcriptomic, proteomic and in silico approaches).

In conclusion, here, we demonstrate that Tel reduces intracellular ERα levels, deregulates E2:ERα signaling and inhibits E2-induced proliferation in BC cells. Remarkably, the findings reported in this work also support the use of selective modulation of intracellular ERα levels as a target to identify new compounds that affect E2:ERα signaling in BC cell proliferation and serve as the basis for future evaluation of Tel repurposing in the treatment of primary and metastatic BC.

## 4. Materials and Methods

### 4.1. Cell Culture and Reagents

17β-Estradiol (E2), Dulbecco’s modified Eagle’s medium (with and without phenol red) and fetal calf serum were purchased from Sigma-Aldrich (St. Louis, MO). A Bradford protein assay kit and anti-mouse and anti-rabbit secondary antibodies were obtained from Bio-Rad (Hercules, CA, USA). Antibodies against ERα (HC-20, rabbit), cyclin D1 (H-295, rabbit), Bcl-2 (C2, mouse), progesterone receptor (C20, rabbit), cathepsin D (H75, rabbit), and pS2 (FL-84, rabbit) were obtained from Santa Cruz Biotechnology (Santa Cruz, CA). Anti-vinculin antibody was purchased from Sigma-Aldrich (St. Louis, MO, USA). Chemiluminescence reagent for Western blotting was obtained from Bio-Rad Laboratories (Hercules, CA, USA). Fulvestrant (i.e., faslodex or ICI 182,780), AZD9496 and brilanestrant (GDC) were purchased from Tocris (Bristol, UK). Telaprevir (VX-950) was purchased from Selleck Chemicals (Houston, TX, USA). All the other products were obtained from Sigma-Aldrich. Analytical- or reagent-grade products were used without further purification.

### 4.2. Western Blot Analysis

Cells were grown in DMEM with phenol red plus 10% fetal calf serum for 24 h and then treated with telaprevir (Tel), fulvestrant (ICI), AZD9496 (AZD) or brilanestrant (GDC) at the indicated doses for the indicated periods. Before E2 stimulation, cells were grown in DMEM without phenol red plus 1% charcoal-stripped fetal calf serum for 24 h; Tel, ICI and cycloheximide (CHX) were added before E2 administration. After treatment, cells were lysed in Yoss Yarden (YY) buffer (50 mM Hepes (pH 7.5), 10% glycerol, 150 mM NaCl, 1% Triton X-100, 1 mM EDTA and 1 mM EGTA) plus protease and phosphatase inhibitors. Western blot analysis was performed by loading 20–30 µg of protein onto SDS-gels. The gels were run, and the proteins were transferred to nitrocellulose membranes with a Turbo-Blot semidry transfer apparatus from Bio-Rad (Hercules, CA, USA). Immunoblotting was carried out by incubating the membranes with 5% milk or bovine serum albumin (60 min), followed by incubation overnight (o.n.) with the indicated antibodies. Secondary antibody incubation was continued for an additional 60 min. Bands were detected using a Chemidoc apparatus from Bio-Rad (Hercules, CA, USA).

### 4.3. Growth Curves

The xCELLigence DP system (ACEA Biosciences, Inc., San Diego, CA, USA) Multi-E-Plate station was used to measure time-dependent responses to E2 and Tel by real-time cell analysis. Each experimental condition was tested in quadruplicate. A detailed description of the instrument and related software has been previously published [59]. The instrument measures the electric impedance of cells on the well surface. The software transforms the measured value of the electric impedance into a nondimensional parameter called the cell index (CI). An increased electric impedance (hence an increased CI) is proportional to an increase in the number of cells. The CI normalized for each well at time 0 was used to follow cell proliferation according to the software manufacturer’s instructions. MCF-7, T47D-1, and BT-474 cells were seeded in growth medium in E-plates 96. After o.n. monitoring of impedance once every 15 min, the medium was changed, and cells were grown in medium containing 1% charcoal-stripped fetal calf serum and Tel in the presence or absence of E2 until the end of the experiment. Cellular impedances were then recorded once every 15 min for a total time of 72 h.

### 4.4. Real-Time Measurement of ERα Transcriptional Activity

MCF-7 cells were stably transfected with a plasmid containing an ERE-nanoluciferase (NLuc)-PEST reporter gene, and real-time measurement of NLuc-PEST expression (i.e., ERα transcriptional activity) was performed as described [30].

### 4.5. In Vitro Binding Assay

A fluorescence polarization (FP) assay was used to measure the binding affinities of telaprevir (Tel) and 17β-estradiol (E2) for recombinant ERα in vitro. The FP assay was performed using a PolarScreen™ ERα Competitor Assay Kit, Green (Thermo Scientific). Measurements were performed according to the manufacturer’s instructions by administering different doses of the test compounds in a final assay reaction that contained ERα (75 nM) and Fluormone ES2 (4 nM) in ERα binding buffer. Each sample was measured in quintuplicate in black 384-well plates, and the experiment was repeated twice. The assay was conducted for 2 h (i.e., to reach steady-state conditions) in the dark at room temperature before the results were read on a Tecan Spark ELISA reader capable of detecting fluorescence polarization.

### 4.6. RNA Isolation and qPCR Analysis

The sequences for gene-specific forward and reverse primers were designed using the OligoPerfect Designer software program (Invitrogen, Carlsbad, CA, USA). The following primers were used: human ERα: 5′-GTGCCTGGCTAGAGATCCTG-3′ (forward) and 5′-AGAGACTTCAGGGTGCTGGA-3′ (reverse) and human GAPDH: 5′-CGAGATCCCTCCAAAATCAA-3′ (forward) and 5′-TGTGGTCATGAGTCCTTCCA-3′ (reverse). Total RNA was extracted from cells using TRIzol reagent (Invitrogen, Carlsbad, Ca, USA) according to the manufacturer’s instructions. To determine gene expression levels, cDNA synthesis and qPCR were performed using the GoTaq 2-step RT-qPCR system (Promega, Madison, MA, USA) in an ABI Prism 7900 HT Sequence Detection System (Applied Biosystems, Foster City, CA, USA) according to the manufacturer’s instructions. Experiments were performed in triplicate. RT-qPCR-based gene array analysis of ERα target gene expression was determined using the PrimePCR Estrogen receptor signaling (SAB Target List) H96 panel (Bio-Rad Laboratories, Hercules, CA, USA) according to the manufacturer’s instructions. Gene expression was normalized to GAPDH mRNA levels.

Gene arrays for estrogen signaling were used according to the manufacturer’s instructions (Bio-Rad).

### 4.7. Bromodeoxyuridine Incorporation Assay

Bromodeoxyuridine (BrdU) was added to the medium in the last 30 min of growth, and the cells were then fixed and permeabilized. Histones were dissociated with 2 M HCl as previously described (Darzynkiewicz and Juan, 2001). BrdU-positive cells were detected with anti-BrdU primary antibody diluted 1:100 (DAKO Cytomatation; Santa Clara, CA, USA) and Alexa488-conjugated anti-mouse antibody diluted 1:100 (Thermo Fisher Scientific; Waltham, MA, USA). Both antibodies were incubated with the cells for 1 h at room temperature in the dark. BrdU fluorescence was measured using a CytoFLEX flow cytometer, and cell cycle analysis was performed with CytExpert v1.2 software (Beckman Coulter, Brea, CA, USA). All samples were counterstained with propidium iodide (PI) for DNA/BrdU biparametric analysis.

### 4.8. Cell Cycle Analysis

After treatments, cells were harvested with trypsin and counted to obtain 10^6^ cells per condition. Then, the cells were centrifuged at 1500 rpm for 5 min at 4 °C, fixed with 1 mL of ice-cold 70% ethanol, and subsequently stained with PI buffer (500 µg/mL PI and 320 µg/mL RNase A in 0.1% Triton X in phosphate-buffered saline). DNA fluorescence was measured using a CytoFLEX flow cytometer (Beckman Coulter, Brea, CA, USA), and cell cycle analysis was performed with CytExpert v1.2 software (Beckman Coulter, Brea, CA, USA).

### 4.9. Statistical Analysis

Statistical analysis was performed using ANOVA (one-way analysis of variance) with Tukey′s post hoc test with InStat version 3 software (GraphPad Software, Inc., San Diego, CA, USA). Densitometric analyses were performed using the freeware ImageJ by quantifying the band intensity of the protein of interest with respect to the intensity of the relative loading control band (i.e., vinculin). The number of experiments is given in the figure text. Data are shown as the mean ± standard deviation. For all analyses, a *p* value < 0.01 indicated significance.

## Supplementary Materials

Supplementary materials can be found at https://www.mdpi.com/1422-0067/21/10/3418/s1. Figure S1: The effect of telaprevir on ERα intracellular levels.
