# Supplementary material for: Unexpected Impact of a Hepatitis C Virus Inhibitor on 17β-Estradiol Signaling in Breast Cancer
Source: Int J Mol Sci. 2020 May 12;21(10):3418. doi: 10.3390/ijms21103418 (PMC7279444; doi:10.3390/ijms21103418)
Supplement: Supplementary file 1 [file ijms-21-03418-s001.pdf]

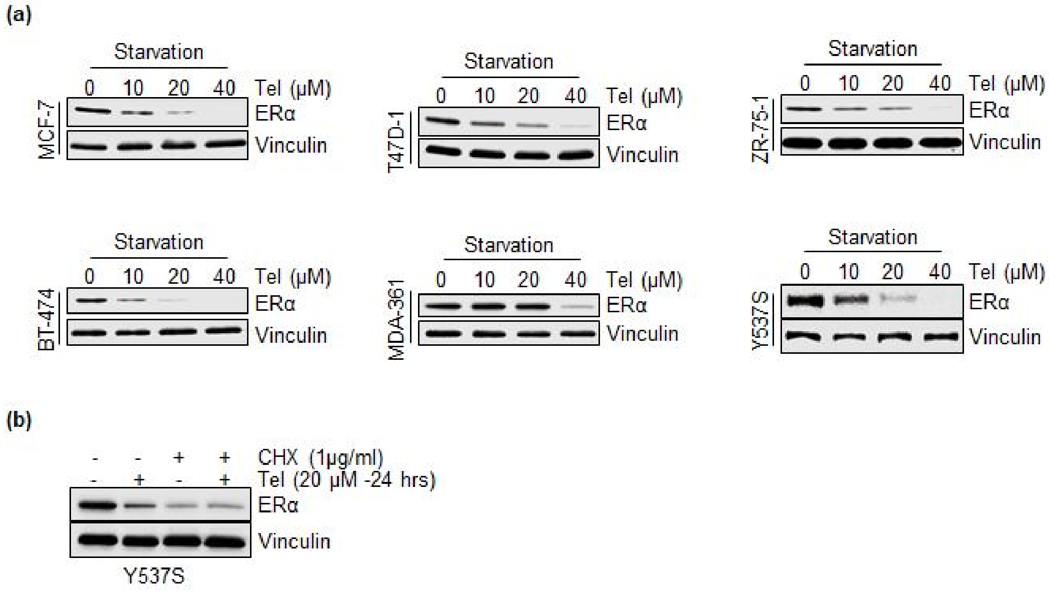

Figure S1. The effect of telaprevir on ERα intracellular levels. (**a**) Western blotting analysis of ERα cellular levels in MCF-7, T47D-1, ZR-75-1, BT-474, MDA-MB-361 and Y537S cells grown in starved condition (DMEM without phenol-red plus 1% charcoal-stripped fetal calf serum). Cells were treated at the indicated doses of telaprevir (Tel) for 24 hours. The loading control was done by evaluating vinculin expression in the same filter. (**b**) Western blotting analysis of pre-formed ERα expression levels in Y537S cells pre-treated with cycloheximide (CHX-1μg/ml) for 6 hours and then treated with Tel (20 μM) for 24 hours. The loading control was done by evaluating vinculin expression in the same filter. Panels a and b show representative blots from at least three independent experiments.
